# Improving resuscitation of pregnant women

**DOI:** 10.1007/s12471-017-0977-0

**Published:** 2017-03-16

**Authors:** H. Lameijer

**Affiliations:** Department of Emergency Medicine, University Medical Centre Groningen, University of Groningen, Groningen, The Netherlands

To the Editor,

In the November edition of this Journal, colleagues Calle et al. described a very interesting case of maternal mortality after cardiac arrest in a woman with situs inversus [1]. While their article mainly focusses on the wrongful delivery of shocks by the AED in this woman, possibly related to her situs inversus, I would like to address two other very important issues in the resuscitation of pregnant women that need to be observed.

Firstly, resuscitation of women who are less than 20 weeks pregnant should not differ from resuscitation in non-pregnant women. However, when resuscitating women who are more than 20 weeks pregnant and who are not in cardiac arrest (as observed in the first lines of the case, where the woman experienced bradycardia with output), a left lateral tilt should be performed to alleviate the aortocaval compression by the gravid uterus, thereby improving venous return and thus increasing preload, stroke volume and cardiac output [2, 3].

Secondly, I would like to address the importance of early perimortem caesarean section (C-section) in cardiac arrest in women who are more than 20 weeks pregnant [3]. A perimortem C‑section is performed to improve maternal outcome, again by alleviating aortocaval pressure caused by the gravid uterus and improving cardiac output, followed by basic and advanced life support measures. Perimortem C‑section should be performed within 4 min of cardiac arrest and should be accomplished within the next 5 min to maximise the maternal cardiac arrest outcome. The classical C‑section approach, in which the natural diastasis of the abdominal muscles can be utilised, can be performed by any doctor who knows how to use a surgical knife [3]. The aorta and vena cava are relatively protected by the uterus and can therefore not be harmed. Also, because there is no output during cardiac arrest, blood loss will be minimal. Furthermore, it should be underlined that perimortem C‑section is performed to improve maternal outcome, however, possible neonatal survival may be an additional benefit.

Whether or not a left lateral tilt or perimortem C‑section may have improved the outcome of the woman presented by Calle et al. is, of course, unknown. Knowledge about how to improve resuscitation of pregnant women, however, is essential for improving their outcomes and should be an element of the medical training of any attendant in the primary or emergency care of pregnant women.
